# Storable bFGF-Releasing Membrane Allowing Continuous Human iPSC Culture

**DOI:** 10.3390/ma14030651

**Published:** 2021-01-31

**Authors:** Ayako Oyane, Hiroko Araki, Maki Nakamura, Yasuhiko Aiki, Yuzuru Ito

**Affiliations:** 1 Nanomaterials Research Institute, National Institute of Advanced Industrial Science and Technology (AIST), Central 5, 1-1-1 Higashi, Tsukuba 305-8565, Ibaraki, Japan; hiroko-araki@aist.go.jp (H.A.); ma-ki-nakamura@aist.go.jp (M.N.); 2 Biotechnology Research Institute for Drug Discovery, National Institute of Advanced Industrial Science and Technology (AIST), Central 5, 1-1-1 Higashi, Tsukuba 305-8565, Ibaraki, Japan; aiki.yasuhiko.ga@u.tsukuba.ac.jp (Y.A.); ito.yuzuru.fe@u.tsukuba.ac.jp (Y.I.)

**Keywords:** basic fibroblast growth factor (bFGF), induced pluripotent stem cell (iPSC), nonwoven fabric, protein adsorption, controlled release, cryopreservation

## Abstract

Basic fibroblast growth factor (bFGF) is a crucial supplement for culture media of human pluripotent stem cells. However, bFGF is extremely unstable under cell culture conditions, which makes frequent (generally every day) medium refreshment requisite. We recently developed a water-floatable, bFGF-releasing membrane via a simple bFGF adsorption process following oxygen plasma treatment by utilizing a polyethylene nonwoven fabric as an adsorbent. This membrane allowed sustained release of bFGF while floating on medium, thereby keeping the bFGF concentration in the medium sufficient for maintaining human-induced pluripotent stem cells (iPSCs) in a proliferative and pluripotent state for as long as 3 days. In this study, lyophilization was applied to the membrane to stabilize bFGF. The sustained bFGF-releasing function of the membrane was kept unchanged even after lyophilization and subsequent cryopreservation at −30 °C for 3 months. The cryopreserved membrane supported proliferation and colony formation of human iPSCs while retaining their viability and pluripotency in a medium-change-free continuous culture for 3 days. The present bFGF-releasing membrane is ready-to-use, storable for at least 3 months, and obviates daily medium refreshment. Therefore, it is a new and more practical bFGF supplement for culture media of human stem cells.

## 1. Introduction

Human pluripotent stem cells such as induced pluripotent stem cells (iPSCs) and embryonic stem cells have enormous potential in basic medicine, drug development, and therapeutic applications [1,2,3]. Culturing of human pluripotent stem cells while maintaining their pluripotency is essential for all of their subsequent applications. In such stem cell maintenance cultures, basic fibroblast growth factor (bFGF) plays an indispensable role; it supports proliferation and colony formation of stem cells while preventing their spontaneous differentiation [4,5,6,7]. Thus, in a standard protocol of human pluripotent stem cell culture, the culture medium is supplemented with bFGF at a concentration from a few nanograms to several tens of nanograms per milliliter to maintain the cell quality. Despite its importance in stem cell culture, bFGF is highly unstable and quickly loses its biological activity in culture conditions at around 37 °C [8,9]. This is the major reason why frequent (generally once every day) medium refreshment is required for stem cell culture, even though it is a significant cost.

To address this issue, new formulations of bFGF supplements for stem cell culture media have been developed, including bFGF-encapsulated biodegradable polymer microbeads [10] and multi-trilayered nanofilms composed of repeating polycation/polyanion/bFGF units [11,12]. These supplements allow sustained release of bFGF in a culture medium of human iPSCs, thereby reducing the frequency of medium refreshment to a biweekly basis. Not only for stem cell maintenance but also for therapeutic and tissue engineering applications, a variety of bFGF-releasing materials have been developed [13,14,15,16]. However, many conventional bFGF-releasing materials consist of degradable components and often contain additives such as heparin (a well-known bFGF stabilizer [9,13,17]) or similar molecules, which may leach into the culture media and affect stem cells directly or indirectly.

Against this background, we recently developed a nondegradable bFGF-releasing membrane without using any additives [18]. We employed a chemically stable, water-floatable polyethylene nonwoven fabric membrane as an adsorbent, and immobilized bFGF on its surface by a simple adsorption process following oxygen plasma treatment. The resulting membrane released bFGF in a sustained manner while floating on medium and enabled medium-change-free continuous culture of human iPSCs for 3 days.

In our previous study, the bFGF-releasing membrane was prepared at each time of use to prevent denaturization of bFGF [18]. That is, a wet membrane just after the bFGF adsorption process was added immediately to the culture medium. This method of use is inconvenient and not practical for laboratory research and development. In this study, we first produced a bFGF-releasing membrane that is ready-to-use and storable under widely available conditions, and second, demonstrated its function as a bFGF supplement for stem cell culture media. To achieve this, lyophilization, a commonly used technique for stabilization of biomolecules [19], was applied to the bFGF-releasing membranes after the bFGF adsorption process. The lyophilized membranes before and after cryopreservation for up to 3 months were evaluated for their bFGF-releasing function in an acellular culture medium by enzyme-linked immunosorbent assay (ELISA). Selected lyophilized membranes were added to culture media of human iPSCs, which were then cultured continuously for 3 days without medium refreshment. The cultured iPSCs were analyzed for their colony appearance, viability, and pluripotency in comparison with standard iPSCs maintained in a bFGF-containing medium with daily medium refreshment.

## 2. Materials and Methods

### 2.1. Materials for Preparation of bFGF-Releasing Membranes

A polyethylene nonwoven fabric (Tyvek^®^ 1073B, DuPont, Wilmington, DE, USA), with a thickness of 180 µm, was used as a bFGF adsorbent. This fabric comprised of randomly oriented microfibers with a diameter of 0.5–10 µm (4 µm on average) and has a microporous structure with a Gurley-Hill porosity of 22 s/100 cc (range: 8–36 s/100 cc) according to the manufacturer’s product information sheet. As a bFGF source, 1 mg/mL human recombinant bFGF (bFGF AF, Katayama Chemical Industries Co., Ltd., Osaka, Japan) was used. For diluting the bFGF source and rinsing the bFGF-adsorbed membranes, phosphate-buffered saline (PBS) (D-PBS(–), FUJIFILM Wako Pure Chemical Corporation, Osaka, Japan) was used.

### 2.2. Preparation of bFGF-Releasing Membranes

**Figure 1:**
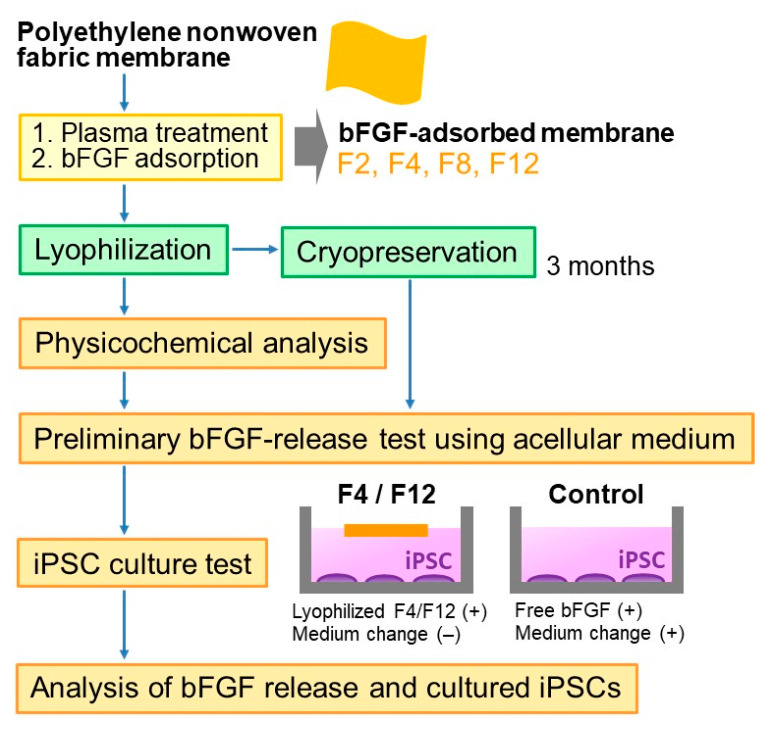
Experimental scheme.

Figure 1 shows the experimental scheme. The bFGF-releasing membranes were prepared according to the method reported previously [18]. First, the polyethylene nonwoven fabric was cut into 10 mm × 10 mm square membranes, ultrasonically washed with ultrapure water and ethanol, and dried in air. The membranes were treated with oxygen plasma at 0.10 W/cm^2^ for 30 s using a compact ion etcher (Model FA-1, Samco Inc., Kyoto, Japan) in oxygen gas (30 Pa) under an electric field operating at 13.56 MHz. Oxygen plasma treatment is a well-known technique to increase water wettability of polymeric materials via the formation of oxygen-containing polar functional groups on their surfaces [20,21]. Such an effect of the plasma treatment was confirmed in our previous study [18]; the contact angle of a water droplet was 119.4 ± 1.6° on the untreated membrane, whereas it was 38.9 ± 5.9° on the plasma-treated membrane.

The plasma-treated membranes were sterilized by exposure to ethylene oxide gas, and then subjected to the bFGF adsorption process. Just before the adsorption process, bFGF solutions with bFGF concentrations of 2, 4, 8, and 12 µg/mL were aseptically prepared by diluting the bFGF source with PBS. The bFGF adsorption process was carried out by immersing the plasma-treated membrane (plasma-treated surface down) in 1 mL of the bFGF solution at 25 °C for 24 h under shaking at 200 rpm with a shaking incubator (DWMaxM BR-104P; TAITEC CORPORATION, Koshigaya, Japan). The membranes removed from bFGF solutions were washed three times with ultrapure water before lyophilization or PBS before preliminary bFGF-release tests. The resulting membranes were denoted as F2, F4, F8, and F12, respectively, according to the bFGF concentration: 2, 4, 8, and 12 µg/mL in the bFGF solution.

### 2.3. Lyophilization and Cryopreservation

The F2, F4, F8, and F12 membranes were frozen at −80 °C and lyophilized for 18 h using a freeze-drier (FDS-1000, TOKYO RIKAKIKAI Co., Ltd., Bunkyo-ku, Japan). A selected lyophilized membrane (F4) was further subjected to cryopreservation in which the membranes were sealed in an aluminum bag and cryopreserved at −30 °C using a medical freezer (FMS-F304G, FUKUSHIMA GALILEI Co. Ltd., Osaka, Japan) for 1 and 3 months.

### 2.4. Physicochemical Analysis

Physicochemical analysis was performed using the lyophilized F12 membrane. A whole picture of the membrane was captured using a digital camera (TG-5; Olympus Corporation, Shinjuku-ku, Japan). The membrane surface was analyzed using a scanning electron microscope (SEM) (Miniscope^®^ TM4000Plus; Hitachi High-Tech Corporation, Minato-ku, Japan), a contact angle meter (Drop Master DM500; Kyowa Interface Science Co., Ltd., Niiza, Japan), a Fourier transform infrared spectrometer (FT-IR) (FT/IR-4700; JASCO Corporation, Hachioji, Japan) equipped with an attenuated total reflection accessory with a monolithic diamond crystal, and an X-ray photoelectron spectrometer (XPS) (PHI 5000 VersaProbe; ULVAC-PHI, Inc., Chigasaki, Japan) with Al Kα radiation (1486.6 eV). An untreated membrane was also analyzed for comparison. Water contact angles were measured 1 s after a droplet (1 µL) of ultrapure water was deposited onto the membrane that was fixed on a flat supporting plate. In XPS analysis, the C1s peak with the binding energy at 284.6 eV was used for charge referencing, and the elemental ratio of the membrane surface was evaluated by PHI MultiPak software.

### 2.5. Preliminary bFGF-Release Test Using Acellular Medium

The F2, F4, F8, and F12 membranes, after lyophilization and subsequent cryopreservation (F4 only), were subjected to a preliminary bFGF-release test using a chemically defined serum-free acellular medium (Essential 6, Thermo Fisher Scientific, Waltham, MA, USA). For comparison, the as-prepared wet F12 membrane (before lyophilization) was also tested.

Each membrane was placed (plasma-treated surface down) over 2 mL of medium in a 24-well culture plate. As a control, medium supplemented with 10 ng/mL bFGF (in place of the membrane) was also tested. After incubation at 37 °C in a humidified 5% CO_2_ atmosphere for various periods up to 72 h, 150-µL aliquots were sampled from the medium and frozen at −80 °C before use in ELISA. The resulting medium loss was compensated by adding identical amounts of the medium. In ELISA, the sampled solutions were diluted with medium and assayed for bFGF using a human FGF basic Quantikine^®^ ELISA kit (R&D Systems, Inc., Minneapolis, MN, USA) according to the manufacturer’s instructions. Standard bFGF solutions were prepared by diluting the bFGF source with the same medium.

### 2.6. bFGF-Release Test Using iPSC-Containing Medium

The lyophilized F4 and F12 membranes and the lyophilized F4 membrane after cryopreservation for 3 months were subjected to the bFGF-release test under culture conditions with human iPSCs (201B7 [22], RIKEN BioResource Research Center, Japan). The use of human iPSCs was approved by the Ethics Committee of AIST (iPS_2016-007), and informed consent was obtained from the donor from which the human iPSC line was generated in accordance with the Declaration of Helsinki. The iPSCs were maintained before use by a standard protocol as described elsewhere [23] using culture medium (TeSR™-E6, STEMCEL-Technologies, Vancouver, BC, Canada) supplemented with 10 ng/mL bFGF and 2 ng/mL transforming growth factor β1 (TGF-β1) (R&D Systems). This medium is referred to as “bFGF-containing medium” and was used in the following cellular experiments unless otherwise specified. A bFGF-free medium was also prepared by adding 2 ng/mL of TGF-β1 only, to the medium (TeSR™-E6).

The iPSCs were seeded (approximately 1.6 × 10^5^ cells/2 mL/well) in a 6-well culture plate that was coated with extracellular matrix proteins (Corning^®^ Matrigel^®^ Growth Factor Reduced Basement Membrane Matrix, Corning Incorporated, Corning, NY, USA) beforehand. After preculture at 37 °C in a humidified 5% CO_2_ atmosphere for 1 day, the bFGF-containing medium was replaced with 2 mL of the bFGF-free medium. Immediately after the medium replacement, each membrane was placed (plasma-treated surface down) over the bFGF-free medium (this time point is defined as Day 0). The iPSCs were cultured in the bFGF-free medium supplemented with the membrane for another 3 days continuously without medium refreshment (referred to as “F4 group” or “F12 group” depending on the membrane type). As a control group, the precultured iPSCs were cultured for 3 days under the standard condition where the bFGF-containing medium was refreshed once a day. After passaging and preculture, the same culture processes were repeated a total of five times (up to the 5th culture).

On Days 1, 2, and 3 in each culture cycle (from 1st to 5th culture) of the F4 and F12 groups, 10 µL aliquots were sampled from the medium. For the control group, sampling of the medium (10 µL) was carried out just before the daily medium refreshment (1 day after the preceding refreshment). The sampled solutions were frozen at −80 °C and used for ELISA following the same protocol as described in the preceding section, except that the diluting fluid was changed to the bFGF-free medium used in this cellular experiment.

### 2.7. Analysis of Cultured iPSCs

The iPSCs obtained on Day 3 in the 1st and 5th cultures of the control, F4, and F12 groups were analyzed in terms of colony appearance, viability, and pluripotency. The colony appearance was observed using a phase contrast microscope (AXIO Vert.A1, Carl Zeiss AG, Oberkochen, Germany). For the viability analysis, the total and viable cell numbers in each well were counted with a cell viability analyzer (Vi-CELL XR, Beckman Coulter, Inc., Brea, CA, USA) after colony dissociation using a cell detachment solution (Accutase^TM^, Innovative Cell Technologies, Inc., San Diego, CA, USA). Cell viability was calculated as the percentage of the viable cell number among the total cell number. The pluripotency of the iPSCs was evaluated by immunofluorescence (Nanog, Oct3/4) and lectin (rBC2LCN) staining following previously reported procedures [18,24]. Prior to staining, the iPSCs colonized on the well were fixed with 4% paraformaldehyde for 10–60 min at 4 °C or room temperature and washed with PBS (DPBS, no calcium, no magnesium, Thermo Fisher Scientific). In the immunofluorescence staining, the iPSCs were incubated at 4 °C overnight with a primary antibody, anti-Oct-3/4 (1:300 dilution, Santa Cruz Biotechnology, Inc., Dalla, TX, USA), or anti-Nanog (1:800 dilution, Cell Signaling Technology, Inc., Danvers, MA, USA), diluted with PBS (Takara Bio, Inc., Kusatsu, Japan) supplemented with 1% bovine serum albumin (BSA) and 5% fetal bovine serum (Thermo Fisher Scientific). Secondary staining was carried out at room temperature for 1 h using a fluorescence dye-conjugated secondary antibody, anti-mouse immunoglobulin M-Alexa488 (1:300 dilution, Thermo Fisher Scientific), or anti-mouse IgG-Alexa488 (1:300 dilution, Thermo Fisher Scientific). In the lectin staining, the iPSCs were incubated at room temperature for 1 h with 10 µg/mL fluorescein isothiocyanate (FITC)-conjugated rBC2LCN (FUJIFILM Wako Pure Chemical Corporation) diluted with PBS (Takara Bio) supplemented with 1% BSA. The iPSCs were counterstained with 4’,6-diamidino-2-phenylindole (DAPI) (DOJINDO LABORATORIES, Kamimashiki-gun, Japan). The stained iPSCs were observed using a fluorescence microscope (BIOREVO BZ-9000; KEYENCE CORPORATION, Osaka, Japan).

### 2.8. Statistical Analysis

In the water contact angle measurement described in Section 2.4, two membranes were tested, and water droplets were deposited on two distinct regions on each membrane surface (four regions in total) to obtain average and standard deviation (SD) values. In the XPS measurement described in Section 2.4, three distinct regions on each membrane surface were analyzed to obtain average and SD values of the N/C elemental ratio of the surface. In the quantitative analysis described in Section 2.5, Section 2.6 and Section 2.7, three membranes were tested for each group to obtain average and SD values. The obtained data were analyzed by Student’s *t*-test, and *p* < 0.05 was considered statistically significant.

## 3. Results and Discussion

### 3.1. Physicochemical Analysis

**Figure 2:**
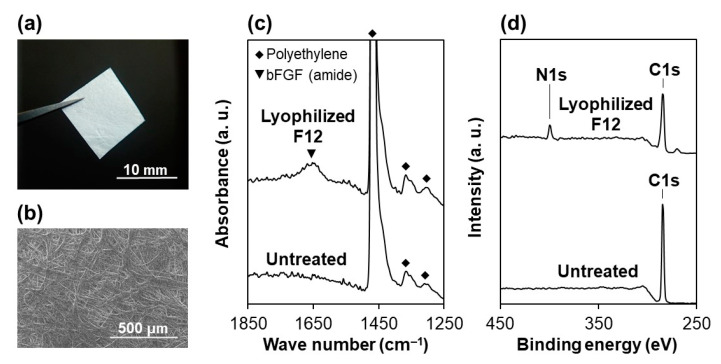
(**a**) Digital camera and (**b**) SEM images of the lyophilized F12 membrane, and (**c**) FT-IR spectra and (**d**) XPS spectra of the surfaces of the untreated and lyophilized F12 membranes.

As depicted in Figure 2a, the lyophilized F12 membrane is a free-standing sheet and easy to handle with tweezers. We observed that this membrane is comprised of randomly oriented nonwoven microfibers and possesses a porous structure (Figure 2b), corresponding to the manufacturer’s product information sheet. The contact angle of a water droplet on this membrane was 33.9 ± 7.7°, which is comparable to that on a plasma-treated membrane (38.9 ± 5.9° [18]). This result indicates that the wetting property of the plasma-treated membrane was retained in the lyophilized F12 membrane. In the FT-IR spectra (Figure 2c), a peak ascribed to bFGF (amide group) was detected for the lyophilized F12 membrane, whereas it was hardly detected for the untreated membrane. The FT-IR spectrum of the lyophilized F12 membrane was similar to that of the non-lyophilized (air-dried) F12 membrane [18]. In the XPS spectra (Figure 2d), the N1s peak, a component element of bFGF, was clearly detected for the lyophilized F12 membrane (N/C elemental ratio = 0.11 ± 0.01), whereas nitrogen was under the detection limit for the untreated membrane (N/C elemental ratio < 0.001). These results suggest the presence of bFGF on the lyophilized F12 membrane.

### 3.2. Preliminary bFGF-Release Test to Examine the Effect of Lyophilization

**Figure 3:**
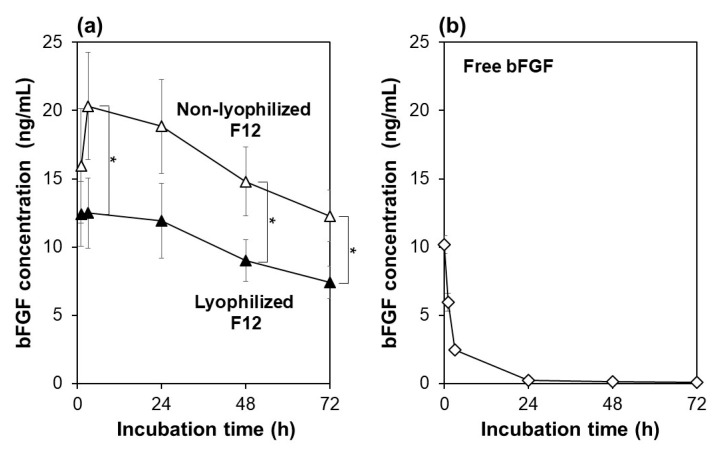
Variations of bFGF concentration with incubation time up to 72 h in the acellular medium supplemented with (**a**) the non-lyophilized (white triangles) or lyophilized (black triangles) F12 membrane, or (**b**) 10 ng/mL of free bFGF (average ± SD; *n* = 3; * *p* < 0.05).

The F12 membrane retained its bFGF-releasing function even after lyophilization, although the net bFGF amount released in the medium decreased to a certain degree as compared to the non-lyophilized membrane. The bFGF-release function of the F12 membranes with and without lyophilization was evaluated using acellular medium. As shown in Figure 3a, both the lyophilized and non-lyophilized F12 membranes released bFGF in the medium and maintained the bFGF concentration in the medium above a few nanograms per milliliter throughout continuous incubation for 72 h. Although both membranes showed similar release profiles of bFGF, the net bFGF concentration in the medium was lower for the lyophilized F12 membrane than for the non-lyophilized membrane (difference was significant at 3, 48, and 72 h). This might be due to the conformational change and aggregation of bFGF molecules adsorbed on the F12 membrane due to lyophilization [25]. Note that “bFGF concentration” in this paper denotes the concentration of bFGF molecules that retained their molecular structure recognizable by the bFGF-specific antibody from the human bFGF ELISA kit.

The lyophilized F12 membrane allowed sustained release of bFGF in the medium as reported for the non-lyophilized membrane [18]. This was confirmed by the control experiment in which the same medium supplemented with free bFGF (10 ng/mL) was incubated under the same condition without membranes for 72 h. As shown in Figure 3b, the bFGF concentration in the medium decreased so quickly in the initial 24 h that the residual rate of bFGF declined from 100% (at 0 h) to 58, 24, and 2% after incubation for 1, 3, and 24 h, respectively. This result agrees with a previous report showing that free bFGF loses most of its biological activity after 24-h incubation at 37 °C [8]. The decrease in residual bFGF concentration in the medium was attributed not only to spontaneous conformational changes of bFGF molecules due to their highly unstable nature [8,9], but also to nonspecific bFGF adsorption onto the well surface [16]. As suggested by a comparison of Figure 3a,b, both the lyophilized and non-lyophilized F12 membranes supplied the medium with ELISA-detectable bFGF molecules in a sustained manner over the incubation period of 72 h. Such sustained bFGF release from the membrane should be due to the limited molecular diffusion within its microporous structure.

### 3.3. Preliminary bFGF-Release Test to Examine the Effect of bFGF Adsorption Conditions

**Figure 4:**
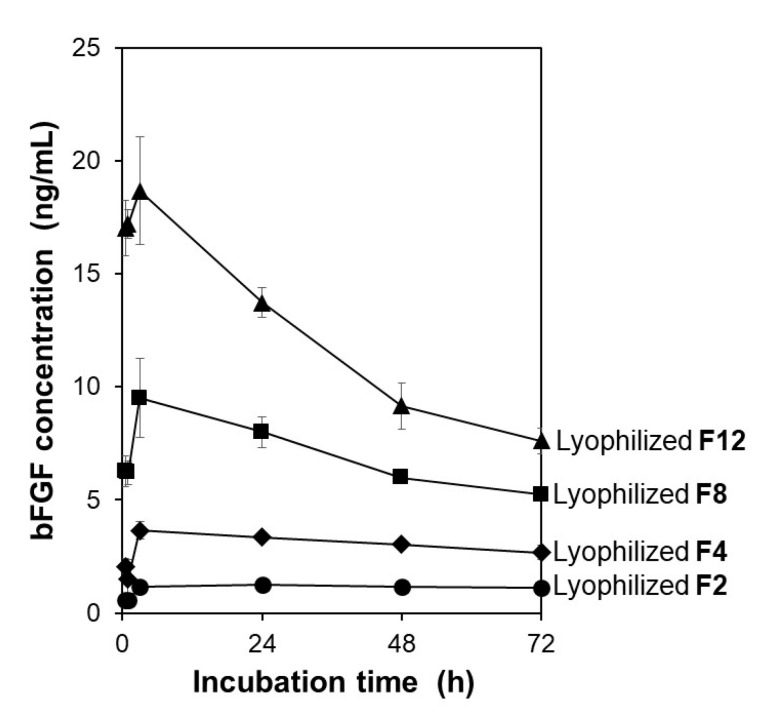
Variations of bFGF concentration with incubation time up to 72 h in the acellular medium supplemented with the lyophilized F2, F4, F8, and F12 membranes (average ± SD; *n* = 3).

The lyophilized membranes exhibited different bFGF-release functions depending on the initial bFGF concentration in the adsorption solution. The F2, F4, F8, and F12 membranes, prepared using adsorption solutions with different bFGF concentrations, were lyophilized and then added to the acellular medium to examine their bFGF release. As shown in Figure 4, all the lyophilized membranes exhibited sustained release of bFGF in the medium similar to the non-lyophilized membranes [18]. The bFGF concentration in the medium increased in the following order: F2 < F4 < F8 < F12 at all sampling points up to 72 h. This was unsurprising considering our previous results showing that the amount of bFGF adsorbed on the membrane increased with increasing initial bFGF concentration in the adsorption solution [18]. Among all the membranes prepared, the lyophilized F4 and F12 membranes were selected and used in subsequent cellular assays.

### 3.4. iPSC Culture Using the Lyophilized Membranes

**Figure 5:**
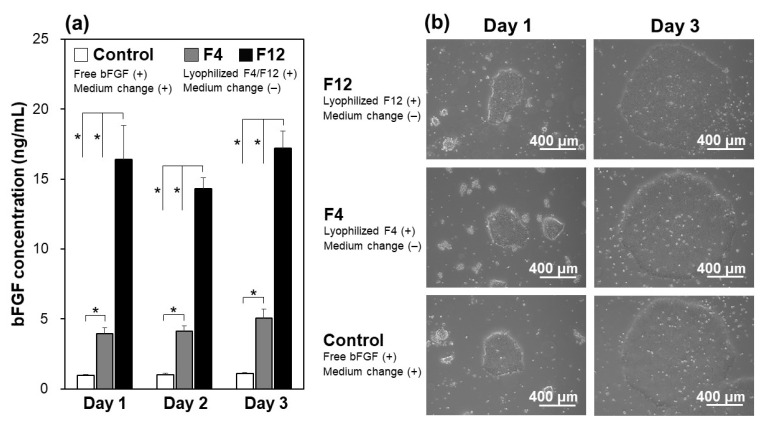
(**a**) Concentrations of bFGF in the culture medium of human iPSCs on Days 1, 2, and 3, in the medium-change-free continuous culture with the lyophilized F4 membrane (F4 group; gray bars) or F12 membrane (F12 group; black bars), or in standard culture using the bFGF-containing medium with daily medium refreshment (control group; white bars) (average + SD; *n* = 3; * *p* < 0.05), and (**b**) phase contrast microscopic images of human iPSCs on Days 1 and 3 in the control, F4, and F12 groups.

The lyophilized F4 and F12 membranes allowed sustained release of bFGF in the culture medium of iPSCs as well as in the acellular medium. In the control group (see white bars in Figure 5a), the residual bFGF concentration in the bFGF-containing medium diminished from 10 ng/mL to approximately 1 ng/mL at the time of daily medium refreshment (1 day after the preceding refreshment with the bFGF-containing medium). In contrast, the bFGF concentration in the medium remained around 5 ng/mL (in the F4 group) or 15 ng/mL (in the F12 group) on Days 1, 2, and 3 during the continuous culture without medium refreshment (see gray and black bars in Figure 5a). This was due to the sustained release of bFGF from the lyophilized membranes as described in Section 3.2. These results suggest that the sustained bFGF release from the lyophilized membranes is hardly influenced by the presence of iPSCs, similar to the non-lyophilized membranes [18]. Note that the residual rate of bFGF in the bFGF-containing medium with iPSCs was higher (approximately 10% on Days 1, 2, and 3, from Figure 5a) than that without cells (approximately 2% at 24 h, from Figure 3b). A similar phenomenon was reported in our previous study [18]. The increased bFGF concentration in the presence of iPSCs was likely caused by autocrine bFGF [26] and/or bFGF-stabilizing molecules (such as heparin [9,13,17]) from iPSCs. Extracellular matrix components and other biomolecules from iPSCs may also increase the residual bFGF concentration through nonspecific adsorption onto the well surface, which reduces bFGF adsorption.

The lyophilized F4 and F12 membranes added to the culture medium of iPSCs supported their proliferation and colony formation in the medium-change-free continuous culture for 3 days. The iPSCs in the F4 and F12 groups proliferated to form large and dense colonies with smooth rounded edges on Day 3 in a similar fashion to those in the control group (Figure 5b). It has been reported that iPSCs cultured in a bFGF-deficient medium are liable to form smaller and more dispersed colonies with jagged edges, implying a change in cellular characteristics (differentiation in most cases) [18]. Such unfavorable colony formation was not observed in either the F4 or F12 groups. Since there was no apparent difference in colony appearance between the F4 and F12 groups (Figure 5b), the lyophilized F4 membrane, which can be produced using less bFGF, was selected and used in subsequent experiments.

**Figure 6:**
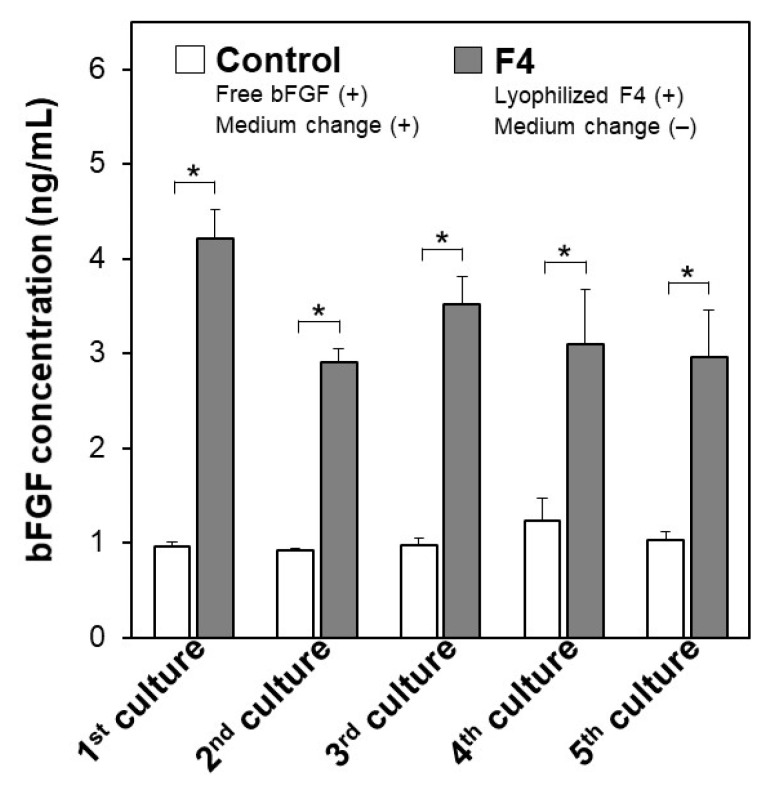
Concentrations of bFGF in the culture medium of human iPSCs, on Day 3 in repeated (up to 5 times) cycles of the medium-change-free continuous culture with the lyophilized F4 membrane (F4 group; gray bars) or of standard culture using bFGF-containing medium with daily medium refreshment (control group; white bars) (average + SD; *n* = 3; * *p* < 0.05).

**Figure 7:**
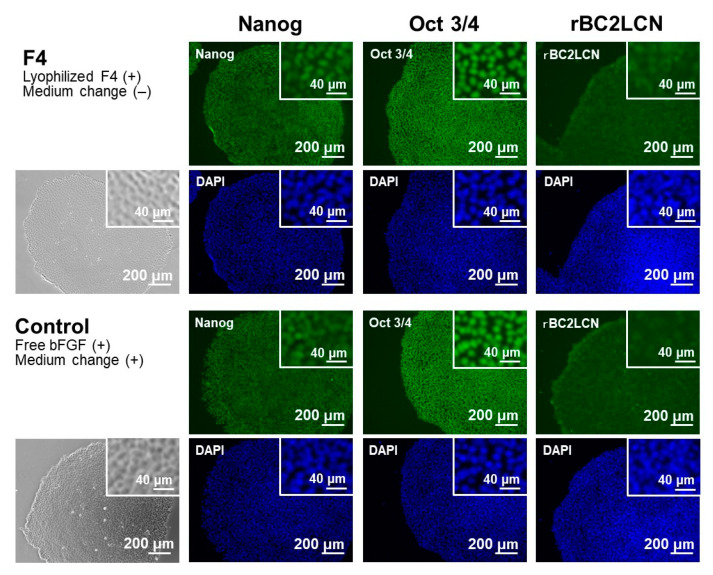
Phase contrast (leftmost column) and fluorescence (right 3 columns) microscopic images of human iPSCs after repeated (5 times) cycles of medium-change-free continuous culture with the lyophilized F4 membrane (F4 group; upper 2 rows) or of standard culture using bFGF-containing medium with daily medium refreshment (control group; lower 2 rows). The iPSCs were stained with FITC (green)-conjugated anti-Nanog, anti-Oct 3/4, and rBC2LCN, and their nuclei were counterstained with DAPI (blue). Insets show higher magnification images.

The pluripotency of iPSCs was retained even after repeated cycles of medium-change-free continuous culture using the lyophilized F4 membrane. Figure 6 shows bFGF concentrations in the culture medium of iPSCs on Day 3 in each culture cycle (from 1st to 5th culture) of the control and F4 groups. In the control group (see white bars in Figure 6), the residual bFGF concentration in the medium on Day 3 was approximately 1 ng/mL in all culture cycles up to the 5th culture, even though the medium was refreshed daily with the bFGF (10 ng/mL)-containing medium. As depicted by the gray bars, the lyophilized F4 membrane maintained a bFGF concentration in the medium above approximately 3 ng/mL even at the end of the continuous culture (on Day 3) in all culture cycles. These results are consistent with those shown in Figure 5a and reconfirm the sustained bFGF-release function of the lyophilized F4 membrane. The pluripotency of the cultured iPSCs was confirmed not only by the colony appearance but also by molecular marker expression. The iPSCs obtained after the 5th culture of the F4 group were in the form of large and dense colonies with smooth rounded edges, and their appearance was comparable to that of the control group (see the leftmost column in Figure 7). These colonies in both the control and F4 groups expressed three pluripotency markers: Nanog, Oct3/4, and rBC2LCN (see the right 3 columns in Figure 7). Oct3/4 is a transcription factor specifically expressed in pluripotent cell lineages such as germ cells and inner cell masses. It is involved in the regulation of pluripotency-specific genes [27]. Nanog along with Oct3/4 is a transcription factor that regulates common downstream genes and contributes to the stabilization of pluripotency [28]. rBC2LCN is a surface marker whose expression has been shown to be highly correlated with pluripotency in human pluripotent stem cells [24]. Since the expression of these pluripotency markers was maintained, we concluded that the iPSCs in both the control and F4 groups maintained pluripotency. In general, stem cell culture is not completed in a single cycle; multiple cycles of culture are required to maintain and multiply stem cells. The results described above demonstrate the efficacy of the lyophilized F4 membrane as a bFGF supplement for stem cell culture media under conditions similar to actual use.

### 3.5. Preliminary bFGF-Release Test to Examine the Effect of Cryopreservation

**Figure 8:**
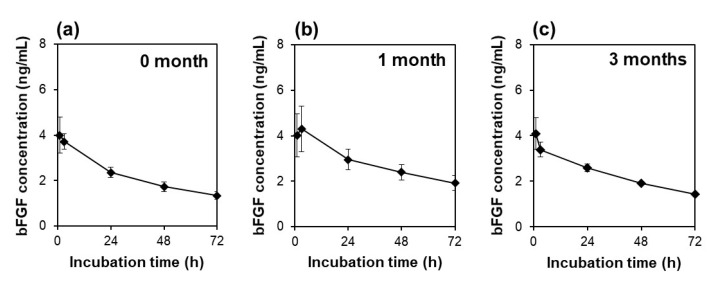
Variations of bFGF concentration with incubation time up to 72 h in acellular medium supplemented with the lyophilized and cryopreserved [(**a**) 0, (**b**) 1, (**c**) 3 months] F4 membrane (average ± SD; *n* = 3).

The sustained bFGF release of the lyophilized F4 membrane did not change for as long as 3 months when cryopreserved at −30 °C. As revealed by a preliminary bFGF-release test using acellular medium, the lyophilized F4 membrane provided similar release profiles of bFGF irrespective of the period of cryopreservation up to 3 months (Figure 8). These results suggest that bFGF molecules adsorbed on the lyophilized F4 membrane were resistant to conformational change in an ordinary medical freezer at −30 °C.

### 3.6. iPSC Culture Using Lyophilized and Cryopreserved Membranes

**Figure 9:**
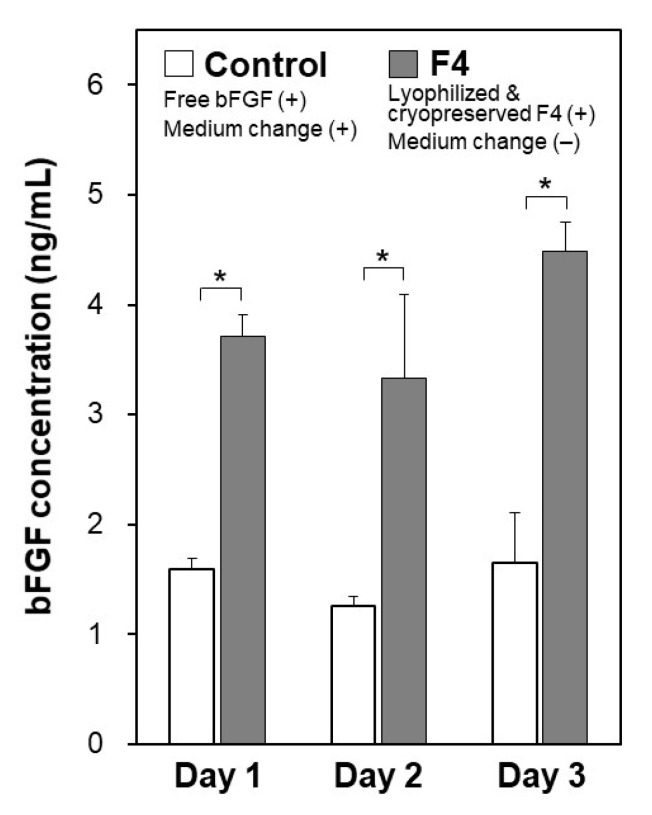
Concentrations of bFGF in the culture medium of human iPSCs on Days 1, 2, and 3 in medium-change-free continuous culture with the lyophilized and cryopreserved (3 months) F4 membrane (F4 group; gray bars) or in standard culture using bFGF-containing medium with daily medium refreshment (control group; white bars) (average + SD; *n* = 3; * *p* < 0.05).

**Figure 10:**
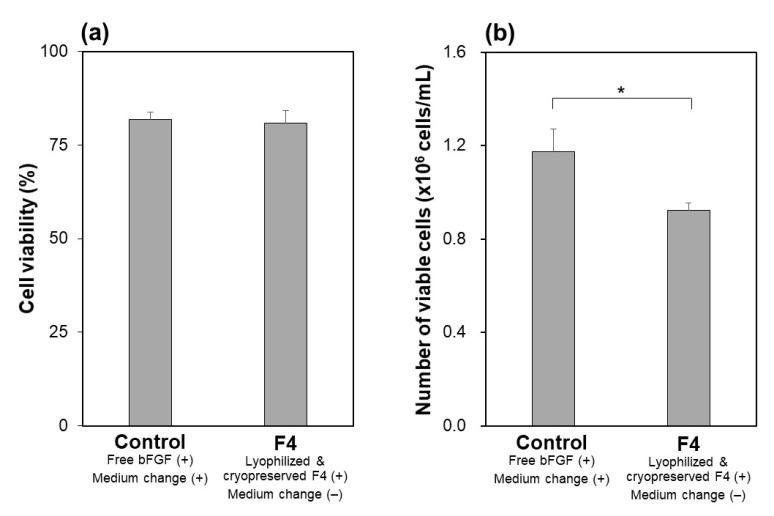
(**a**) Viability of iPSCs and (**b**) relative number of viable iPSCs on Day 3 in medium-change-free continuous culture with the lyophilized and cryopreserved (3 months) F4 membrane (F4 group) or in standard culture using bFGF-containing medium with daily medium refreshment (control group) (average + SD; *n* = 3; * *p* < 0.05).

**Figure 11:**
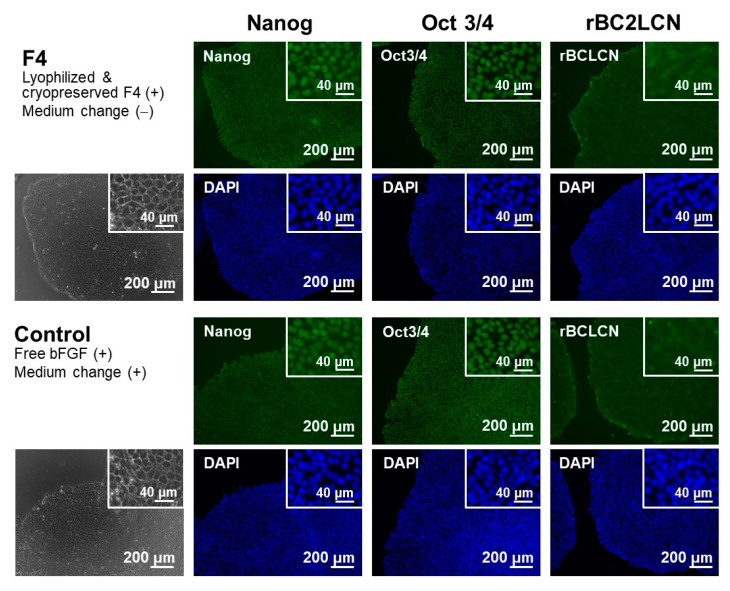
Phase contrast (leftmost column) and fluorescence (right 3 columns) microscopic images of human iPSCs on Day 3 in medium-change-free continuous culture with the lyophilized and cryopreserved (3 months) F4 membrane (F4 group; upper 2 rows) or in standard culture using bFGF-containing medium with daily medium refreshment (control group; lower 2 rows). The iPSCs were stained with FITC (green)-conjugated anti-Nanog, anti-Oct 3/4, and rBC2LCN, and their nuclei were counterstained with DAPI (blue). Insets show higher magnification images.

The function of the lyophilized F4 membrane as a bFGF supplement for stem cell culture media was retained even after cryopreservation at −30 °C for 3 months. As suggested by the higher bFGF concentration for the F4 group than for the control group (Figure 9), the lyophilized F4 membrane exhibited sustained bFGF release in culture medium with iPSCs after cryopreservation for 3 months. As depicted by gray bars in Figure 9, the bFGF concentration for the F4 group (lyophilized F4 membrane after cryopreservation) was 3–5 ng/mL, which was equivalent to the concentration range achieved by the lyophilized F4 membrane without cryopreservation (see gray bars in Figure 5a). The iPSCs on Day 3 in the control and F4 groups were comparable in viability (Figure 10a) and colony appearance (see leftmost images in Figure 11). However, the number of viable iPSCs in the F4 group was significantly smaller than that in the control group (Figure 10b), suggesting a slower cell proliferation rate in the F4 group. This is likely due to the lower frequency of medium refreshment in the F4 group, which causes cellular waste accumulation and/or shortages of certain nutrients (other than bFGF) in the medium. As visualized in Figure 11, the iPSCs in the F4 group expressed all of the pluripotency markers (Nanog, Oct3/4, and rBC2LCN) tested in this study. There was no apparent difference in pluripotency marker expression levels between the control and F4 groups. It is apparent from these results that the lyophilized F4 membrane can be stored in a freezer (−30 °C) for at least 3 months before use as a bFGF supplement to the culture medium of iPSCs.

### 3.7. Mechanism and Function

We successfully prepared bFGF-releasing membranes that can be cryopreserved for months via lyophilization of bFGF-adsorbed nonwoven fabric membranes. In the bFGF adsorption process, a relatively large amount of bFGF (1.74 ± 0.08 µg/cm^2^ for the F4 membrane [18]) was adsorbed on the plasma-treated membrane surface, taking advantage of the large surface area because of its microfibrous structure (Figure 2b) and the improved surface wettability due to the prior oxygen plasma treatment. According to our previous report [18], bFGF adsorption on the plasma-treated membrane can be explained by the Langmuir adsorption model. We consider that bFGF adsorption is mediated mainly by physisorption caused by electrostatic interactions as well as van der Waals interactions between bFGF molecules and the plasma-treated membrane surface. In the subsequent lyophilization process, the bFGF molecules adsorbed on the membrane surface are stabilized by releasing moisture while retaining their biological effects on iPSCs, although some of them undergo conformational change and aggregation [25] as suggested by the results shown in Figure 3a. When the lyophilized membrane is added to the culture medium, the bFGF molecules on the membrane should be desorbed and released into the medium as a result of the altered equilibrium from the adsorption conditions (Le Chatelier’s principle). Exchanging reactions between the bFGF molecules adsorbed on the membrane and other biomolecules (transferrin, insulin, etc.,) in the medium should also be involved in the bFGF desorption from the membrane surface. Since mass transfer is restricted within the microporous structure of the membrane, bFGF desorption from the membrane surface proceeds gradually, which should be responsible for the sustained bFGF release by the membrane. In the culture medium of iPSCs, the lyophilized membrane released bFGF in a sustained manner, thereby keeping the iPSCs in a proliferative and pluripotent state during the medium-change-continuous culture (Figure 5, Figure 6 and Figure 7), similar to the non-lyophilized membrane [18]. Furthermore, the functioning of the lyophilized membrane was unchanged even after cryopreservation for 3 months (Figure 8, Figure 9, Figure 10 and Figure 11).

### 3.8. Advantages of the Present bFGF-Releasing Membrane

Nonwoven fabrics have long been used as biomaterials such as wound dressings, artificial dura maters, and medical gauzes and masks. Recently, nonwoven fabrics have been extensively studied for new biological applications (e.g., as drug delivery carriers and tissue engineering scaffolds) [29,30,31]. In this paper, we report the application of a nonwoven fabric membrane as a bFGF-releasing carrier for continuous human iPSC culture. A primary advantage of the present bFGF-releasing membrane over conventional bFGF-releasing materials is its component simplicity: bFGF and non-degradable adsorbent without any additives. It is well-known that lyoprotectants and cryoprotectants can protect proteins during freeze-drying and freeze-thawing processes, respectively [19]. For bFGF, molecules such as sucrose, lactose, mannitol, glucose, polyethylene glycol, and glucose have been reported as effective lyoprotectants/cryoprotectants [13,25]. It is also known that certain types of molecules such as heparin, similar glucosaminoglycans, and heparin-like synthetic polymers can stabilize bFGF by binding to the heparin-binding site of bFGF [13,17]. Conventional bFGF-releasing materials often contain these additives to ensure their sustained bFGF release. In addition, a large majority of conventional bFGF-releasing materials consist of degradable components. When such bFGF-releasing materials are used as bFGF supplements for stem culture media, degradation products and/or additives may leach into culture media, thereby affecting stem cells. For instance, a multi-trilayered nanofilm composed of repeating polycation/polyanion/bFGF units (bFGF+HEP MNF in [11]) has been reported to release bFGF in a similar fashion to the present bFGF-releasing membrane; the concentration of ELISA-detectable bFGF in PBS (37 °C) maximized after 8 h of incubation and decreased by 40% after 72 h. However, this nanofilm contained not only bFGF but also poly(*β*-amino ester), collagen, and heparin as the components. The present bFGF-releasing membrane is composed of a non-degradable polyethylene nonwoven fabric with adsorbed bFGF and is free of any additives. Throughout the preparation process, any reagents that may raise safety concerns, such as organic solvents or animal-derived materials, are not utilized. Moreover, the present bFGF-releasing membrane is floatable and non-dispersible in a culture medium, which is different from particulate materials and enables non-contact bFGF supply to adherent stem cells and easy membrane removal from the culture system after use. This is an additional advantage of the present bFGF-releasing membrane for use as a bFGF supplement for stem cell culture media.

### 3.9. Potential of the Present bFGF-Releasing Membrane

The present bFGF-releasing membrane has a potential as a new source of bFGF supplementation for culture media of human stem cells. In a standard protocol of human stem cell culture, medium supplemented with bFGF is refreshed daily because of the unstable nature of bFGF. This unstable nature was confirmed by our observation that the concentration of bFGF (ELISA-detectable bFGF) decreased sharply from 10 to 0.2 ng/mL (approximately a 98% reduction) within 24 h in the acellular medium (Figure 3b). In contrast, the lyophilized membranes and those subsequently cryopreserved for 3 months maintained the bFGF concentration in the medium above a few nanograms per milliliter for as long as 72 h via the sustained release of bFGF (Figure 3a, Figure 4 and Figure 8). Thus, the addition of these membranes to the culture medium of human iPSCs enabled medium-change-free continuous culture for 3 days during which iPSCs formed fine colonies while retaining their pluripotency (Figure 5, Figure 7 and Figure 11). We reported similar results for membranes without lyophilization as well [18]. However, the non-lyophilized membranes lack storage stability and need to be used immediately after the adsorption process. The lyophilized membranes are storable for at least 3 months and hence have added practical value compared to the non-lyophilized membranes. Although longer stability tests at different temperatures are necessary, the results obtained here suggest the production capability of the storable and ready-to-use bFGF-releasing membranes. Storable and ready-to-use bFGF-releasing membranes offer many advantages for not only users but also manufacturers, e.g., usability, ease of quality control, economic viability, and distributability to the world market. The present bFGF-releasing membrane is ready-to-use, storable for at least 3 months, and has proven efficacy for reducing the frequency of medium refreshment in the maintenance culture of human iPSCs. Hence, it would contribute to stem cell-based research and development by streamlining the maintenance of human stem cells as a new and more practical bFGF supplement for culture media.

## 4. Conclusions

Lyophilization was effective in stabilizing bFGF-adsorbed nonwoven fabric membranes without impairing their function as a bFGF supplement for stem cell culture media. The lyophilized membrane floated on the medium and released bFGF in a sustained manner, thereby maintaining the bFGF concentration in the medium sufficient for stem cell culture for as long as 3 days. The lyophilized membrane supported proliferation and colony formation of human iPSCs in a medium-change-free continuous culture for 3 days. The resulting iPSCs retained pluripotency similar to standard iPSCs maintained in bFGF-containing medium that was refreshed daily. So, the lyophilized membrane was effective in reducing the frequency of medium refreshment in a maintenance culture of iPSCs. The functioning of the lyophilized membrane was unchanged even after the membrane was subsequently cryopreserved for 3 months. Since the lyophilized membrane is ready-to-use and storable for at least 3 months, it can serve as a new and more practical bFGF supplement for stem cell culture media.

## Data Availability

Data is contained within the article.
